# Age-Stratified Median Effective Dose of a Continuous Remimazolam Maintenance Infusion for Moderate Sedation During Spinal Anesthesia: A Prospective Up-and-Down Dose-Finding Study

**DOI:** 10.3390/medicina62101937

**Published:** 2026-10-08

**Authors:** Se-Ung Park, Yeon Ju Kim, Min Chul Yang, Chan-Hye Park, Hyungtae Kim, Ha-Jung Kim, Won Uk Koh

**Affiliations:** 1 Department of Anesthesiology and Pain Medicine, Asan Medical Center, University of Ulsan College of Medicine, 88 Olympic-Ro 43-Gil, Songpa-Gu, Seoul 05505, Republic of Korea; d250594@amc.seoul.kr (S.-U.P.); yjans@amc.seoul.kr (Y.J.K.); ky3544@gmail.com (M.C.Y.); usgra@amc.seoul.kr (H.K.); 2 Department of Anesthesiology and Pain Medicine, Seoul Medical Center, 156 Sinnae-Ro, Jungnang-Gu, Seoul 02053, Republic of Korea

**Keywords:** remimazolam, conscious sedation, spinal anesthesia, aged, dose–response relationship, drug, up-and-down design

## Abstract

*Background and Objectives*: Remimazolam is an ultra-short-acting benzodiazepine suitable for continuous infusion; however, evidence regarding its maintenance infusion rate for moderate sedation during spinal anesthesia across age strata is limited. We aimed to estimate and compare the continuous maintenance median effective dose (ED_50_) of remimazolam across three prospectively defined age groups. *Materials and Methods*: In this prospective, single-center dose-finding study (ClinicalTrials.gov NCT05379777), 60 patients undergoing lower-extremity orthopedic surgery under spinal anesthesia were stratified into Group A (<60 years), Group B (60–74 years), and Group C (≥75 years) (*n* = 20 each). After a loading infusion (1.0 mg/kg/h) to achieve moderate sedation (Modified Observer’s Assessment of Alertness/Sedation [mOAA/S] score ≤ 3), maintenance rates were assigned using Dixon’s up-and-down method; success was defined as maintaining mOAA/S ≤ 3 until the end of surgery without dose titration. The maintenance ED_50_ was estimated using centered isotonic regression with 90% confidence intervals (CIs). Safety outcomes were evaluated in all patients. *Results*: Sedation was maintained at the assigned rate in 13, 13, and 14 patients in Groups A, B, and C, respectively, and all failures were managed by upward titration. The maintenance ED_50_ was 0.238 (90% CI, 0.152–0.336), 0.233 (90% CI, 0.170–0.301), and 0.178 (90% CI, 0.092–0.245) mg/kg/h in Groups A, B, and C, respectively; the CIs overlapped, and no statistically significant difference was observed. Hypotension requiring vasopressors occurred in 25.0% and respiratory depression requiring airway management in 23.3% of patients, without significant intergroup differences; no patient had peripheral oxygen saturation < 90% or required flumazenil. *Conclusions*: The maintenance ED_50_ of remimazolam for moderate sedation during spinal anesthesia was numerically lower in patients aged ≥75 years, without a statistically significant difference among age groups. A conservative initial maintenance rate may be considered in this age group; this hypothesis-generating suggestion requires confirmation in adequately powered studies.

## 1. Introduction

Spinal anesthesia is a widely used and effective anesthetic technique for lower-extremity orthopedic surgeries. Although it provides reliable sensory and motor blockade, many patients experience significant intraoperative anxiety, fear, and discomfort. Therefore, appropriate intraoperative sedation is highly recommended to improve patient comfort and surgical compliance [1,2]. This clinical need is particularly prominent in geriatric patients, who have reduced physiological reserves and increased vulnerability to perioperative stress [3]. However, sedatives used during regional anesthesia can cause dose-dependent respiratory depression, hypotension, bradycardia, or delayed emergence, particularly in elderly patients [4].

Remimazolam is an ultra-short-acting γ-aminobutyric acid type A receptor agonist that has been proposed as an alternative sedative in this setting [5]. It contains an ester-linked side chain, which allows it to be rapidly hydrolyzed, mainly by carboxylesterase 1 in the liver, into an inactive metabolite (CNS7054) [6]. This pharmacological profile is associated with a rapid onset, a short and predictable context-sensitive half-life, and minimal drug accumulation even after prolonged continuous infusion [6]. Previous studies have reported a lower incidence of hypotension and respiratory depression with remimazolam than with propofol in some clinical settings, although these findings vary across populations [7,8]. In addition, its sedative effect can be reversed by flumazenil [8].

Despite these pharmacological characteristics, the optimal dosing regimen of remimazolam for continuous maintenance sedation during spinal anesthesia remains unclear. With advancing age, progressive changes in central nervous system sensitivity and pharmacokinetic clearance can alter drug requirements [9]. Previous studies have examined age-related differences in remimazolam requirements, including the dose required for loss of consciousness during general anesthesia induction across age groups [9,10], and the age-related response in older patients undergoing orthopedic surgery [11]. However, data on continuous maintenance infusion to maintain moderate sedation during spinal anesthesia are scarce. In clinical practice, continuous infusion is often preferred over intermittent boluses to maintain a stable and safe sedative depth. Furthermore, because the geriatric population is heterogeneous, subdividing older patients into young–old and old–old cohorts may help avoid both under-sedation and accidental drug accumulation [11].

Therefore, the primary aim of this prospective study was to determine and compare the continuous maintenance median effective dose (ED_50_) of remimazolam for moderate sedation during spinal anesthesia across three prospectively stratified age groups: non-elderly (aged <60 years, Group A), young–old (60–74 years, Group B), and old–old (aged ≥75 years, Group C) patients undergoing lower-extremity orthopedic surgery. By employing a statistically sequential up-and-down allocation design combined with centered isotonic regression (CIR) [12], we aimed to estimate and compare age-specific continuous maintenance ED_50_ values of remimazolam. To our knowledge, this is the first prospective study to estimate the continuous maintenance ED_50_ of remimazolam for moderate sedation during spinal anesthesia.

## 2. Materials and Methods

### 2.1. Study Design and Ethical Approval

**Figure 1:**
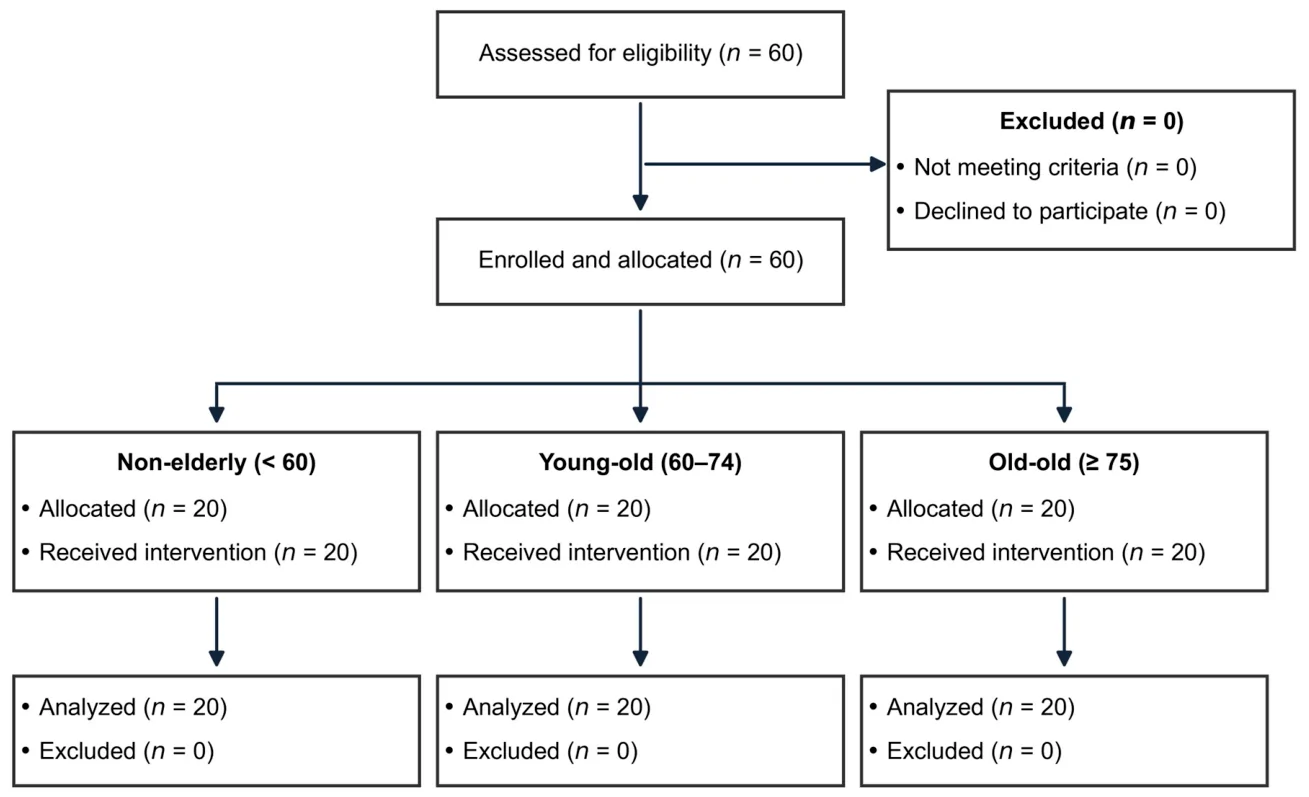
Flow diagram of patient recruitment and enrollment. Flowchart illustrating the screening, allocation, and tracking of participants across the three predefined age cohorts: Group A (non-elderly, <60 years, *n* = 20), Group B (young–old, 60–74 years, *n* = 20), and Group C (old–old, ≥75 years, *n* = 20). All 60 enrolled participants completed the study protocol without crossover, exclusion, or loss to follow-up.

This prospective, single-center, age-stratified dose-finding study was conducted at Asan Medical Center, Republic of Korea. The study protocol was approved by the Institutional Review Board (IRB No. 2021-1869, approved on 31 December 2021). Patient screening was initiated on 10 May 2022, and the first patient was enrolled on 11 May 2022. Public trial registration was submitted to ClinicalTrials.gov (NCT05379777) on 13 May 2022 (first posted on 18 May 2022). Consequently, trial registration was submitted 2 days after the first patient enrollment, during which two patients, both in Group C, were enrolled. Both patients were managed according to the IRB-approved protocol and were included in the analysis because their exclusion would have disrupted the sequential up-and-down allocation in Group C. Patient enrollment and data collection were conducted between May 2022 and October 2022, and written informed consent was obtained from all patients prior to enrollment. This study was conducted in accordance with the Declaration of Helsinki (2013). This manuscript was prepared following the Transparent Reporting of Evaluations with Nonrandomized Designs statement checklist, and participant flow is presented in Figure 1.

### 2.2. Patients

Patient selection criteria included age ≥ 20 years, American Society of Anesthesiologists (ASA) physical status of I to III, and scheduling for lower-extremity orthopedic surgery (including knee and hip procedures) under spinal anesthesia. Exclusion criteria were patient refusal, hypersensitivity to benzodiazepines or flumazenil, severe hepatic or renal dysfunction, a history of substance or alcohol abuse, chronic use of psychotropic or anticonvulsant medications, severe obstructive sleep apnea, expected difficult airway, and any contraindication to spinal anesthesia. Eligible patients were prospectively stratified into three independent age groups: non-elderly (<60 years, Group A, *n* = 20), young–old (60–74 years, Group B, *n* = 20), and old–old (≥75 years, Group C, *n* = 20), representing a total of 60 subjects [11,13]. Procedural and perioperative variables were recorded for all participants, including the type of surgical procedure, anesthesia duration, surgical duration, total sedation duration, spinal bupivacaine and intrathecal fentanyl doses, achieved dermatomal sensory blockade levels, adjunctive regional analgesic techniques (fascia iliaca compartment block, quadratus lumborum block, femoral nerve block, or epidural supplementations).

### 2.3. Spinal Anesthesia and Sedation Protocol

Upon arrival in the operating room, standard monitoring including electrocardiography, non-invasive blood pressure, and pulse oximetry was established. Continuous electroencephalographic Patient State Index (PSI; SedLine, Masimo Corporation, Irvine, CA, USA) monitoring was applied to evaluate the depth of sedation. Supplemental oxygen was delivered via a facial mask at 4–6 L/min throughout the procedure with end-tidal carbon dioxide monitoring. Spinal anesthesia was performed at the L3–4 or L4–5 interspace, and intrathecal 0.5% hyperbaric bupivacaine (12–15 mg) combined with fentanyl (15 µg) was administered to achieve a target sensory blockade level at or above T10. Before initiating remimazolam administration, a stable sensory block level at or above T10 was confirmed via cold or pinprick sensation. For intraoperative and postoperative pain management, procedure-specific regional analgesia was routinely integrated with spinal anesthesia. Hip procedures received an ultrasound-guided fascia iliaca compartment block or quadratus lumborum block, while complex knee procedures received an adjunctive ultrasound-guided femoral nerve block or epidural supplementation. All perioperative management strategies and adjunctive regional analgesic techniques were implemented in accordance with our institutional clinical protocols based on the specific type of surgery and anticipated surgical duration.

Sedation induction was initiated with a loading infusion rate of 1 mg/kg/h of remimazolam besylate (Byfavo; Hana Pharm Co., Ltd., Seoul, Republic of Korea). Loss of responsiveness (LOR) was defined as reaching a target moderate sedation depth of Modified Observer’s Assessment of Alertness/Sedation (mOAA/S) score of ≤3 (i.e., responding only after name is called loudly and/or repeatedly [score 3] or after mild prodding/shaking [score 2]). Because an mOAA/S score of 3 includes a response only after the name is called loudly or repeatedly, this threshold represents the deeper end of moderate sedation, adjacent to deep sedation. Time to LOR (defined as the elapsed time in seconds from the initiation of loading infusion until LOR) and the cumulative loading dose required to achieve LOR (mg) were recorded for each patient. Immediately upon reaching LOR, the loading infusion was stopped, and continuous maintenance infusion was initiated at the predetermined maintenance dose assigned to the patient. During the maintenance phase, the mOAA/S score was assessed every 5 min. If a patient exhibited inadequate sedation (mOAA/S score 4 or 5) during the maintenance phase, the maintenance infusion rate was titrated upward in increments of 0.1–0.2 mg/kg/h until adequate sedation was restored. Any patient requiring such incremental dose adjustments was classified as a sedation failure for the primary up-and-down analysis.

Continuous remimazolam maintenance infusion was discontinued immediately upon completion of the surgical procedure (defined as the start of subcutaneous tissue closure). Thereafter, recovery of responsiveness (ROR) was monitored during the emergence phase, defined as the precise moment the patient opened their eyes in response to a normal verbal command. Time to ROR was recorded as the elapsed time in seconds from drug cessation until eye-opening.

### 2.4. Up-and-Down Dose-Finding and Sequential Allocation

The maintenance infusion rate of remimazolam was determined sequentially for each age cohort using Dixon’s classical up-and-down method. The initial maintenance infusion rate for the first patient in each cohort was set at 1.0 mg/kg/h. Success was defined as maintaining stable moderate sedation (mOAA/S score ≤ 3) continuously from the end of the induction phase until the completion of surgery without requiring any dose titration. Failure was defined as an mOAA/S score above 3 requiring dose titration. Dose adjustment gradients were defined dynamically to optimize the efficiency of the up-and-down random walk: from 1.0 mg/kg/h down to 0.6 mg/kg/h, changes were made in steps of 0.2 mg/kg/h; below 0.6 mg/kg/h, changes were made in steps of 0.1 mg/kg/h. If sedation succeeded in the previous patient, the maintenance infusion rate for the next patient was decreased by one step; if it failed, the rate was increased by one step. Enrollment in each cohort was planned to continue until 20 patients had been enrolled and six independent failure-to-success crossover pairs had been obtained, and was completed at the prespecified sample size of 20 patients per cohort. Because of the sequential up-and-down design, the study was conducted in an open-label manner; the attending anesthesiologist who administered the remimazolam infusion also assessed sedation depth and was aware of the assigned infusion rate.

### 2.5. Patient State Index Monitoring and Pharmacokinetic Simulation of Effect-Site Concentration

The PSI values were analyzed at baseline (defined as immediately before the initiation of the remimazolam loading infusion) and three post-administration clinical landmarks: LOR, drug cessation, and ROR. To determine the drug concentration at the site of action, the effect-site concentration (C_e_) of remimazolam was simulated post hoc using the Asan Pump software (version 2.1.3, Bionet Co., Ltd., Seoul, Republic of Korea). The simulation was based on the pharmacokinetic and pharmacodynamic parameters from the established Schüttler three-compartment model [14,15], applying a blood–brain equilibration rate constant of 0.27 min^−1^. Simulated C_e_ values (µg/mL) were calculated at each designated clinical landmark (LOR, drug cessation, and ROR, where applicable) based on individual body weight and precise infusion history. No C_e_ values were calculated at baseline as drug administration had not yet commenced.

### 2.6. Safety Variables

All patients routinely received supplemental oxygen via facial mask during the procedure. Safety evaluations included continuous monitoring of hemodynamic and respiratory parameters. Hypotension was defined as a mean blood pressure < 60 mmHg or a decrease of more than 20% from baseline, and was treated with vasopressors (ephedrine or phenylephrine). Bradycardia was defined as a heart rate < 50 beats/min and was treated with atropine. Respiratory depression was defined as a respiratory rate < 6 breaths/min or peripheral oxygen saturation (SpO_2_) < 90%; transient mild desaturation (SpO_2_ ≤ 95%) was also recorded. When respiratory depression occurred, initial management consisted of immediate airway maneuvers (head tilt or chin lift) and increased oxygen flow. If respiratory depression persisted despite these, oral airway insertion (or nasal airway insertion) was performed. For persistent cases despite these airway interventions, bag-mask ventilation was initiated, and flumazenil reversal was considered if necessary.

### 2.7. Statistical Analysis

The sample size of 20 patients per group was chosen on the basis of sample sizes commonly used in up-and-down studies targeting the ED_50_ in anesthesiology [12,16]; no study-specific simulation was performed.

The maintenance ED_50_ of remimazolam was estimated independently for each age group using CIR as the primary nonparametric estimator, and analytic 90% confidence intervals (CIs) were calculated [12]. Additionally, to provide clinical reference for higher-percentile effective doses, exploratory maintenance 80%, 85%, and 90% effective doses (ED_80_, ED_85_, and ED_90_) with their corresponding analytic 90% CIs were estimated using the primary CIR framework [12]. As sensitivity analyses, two-parameter log-logistic regression models were fitted using 2000 percentile bootstrap replicates specifically to evaluate the robustness of the primary ED_50_ estimates [16]. Given the inherent median-clustering property of Dixon’s up-and-down design, where data points are concentrated around the median, these higher-percentile effective doses (ED_80_, ED_85_, and ED_90_) were reported as exploratory estimates only, and their relatively wide confidence intervals were interpreted with caution. The primary safety outcomes were hypotension requiring vasopressor support and respiratory depression requiring airway management; these and all other intraoperative adverse events were evaluated across the full cohort (N = 60; *n* = 20 per group), with risk differences relative to Group A and their 95% confidence intervals (Newcombe hybrid score method) reported alongside exact *p* values. Exploratory secondary pharmacodynamic parameters (including PSI and simulated C_e_ at key landmarks) were summarized for recorded cases at each landmark, excluding missing data or unreached landmarks. Because these exploratory comparisons were not adjusted for multiplicity, *p* values near 0.05 were interpreted as signals rather than findings.

Continuous variables were assessed for normality using the Shapiro–Wilk test; normally distributed data are presented as mean ± standard deviation and compared using one-way analysis of variance, whereas non-normally distributed data or small-sample subsets are presented as median [interquartile range] and compared using the Kruskal–Wallis test. Categorical variables were analyzed using the chi-square test or Fisher’s exact test. A two-sided *p* < 0.05 was considered statistically significant. All analyses were performed in R version 4.2.1 (R Foundation for Statistical Computing, Vienna, Austria) using the *cir*, *upndown*, *binom*, and *boot* packages. A fixed random seed (20211869) was set for the bootstrap resampling to ensure computational reproducibility.

## 3. Results

### 3.1. Participant Flow and Baseline Characteristics

A total of 60 patients (20 per age group) were assessed, enrolled, and completed the study protocol without exclusion or loss to follow-up (Figure 1). Baseline demographic and clinical characteristics are summarized in Table 1. Age and height differed significantly across the groups (*p* < 0.001 and *p* = 0.001, respectively). The proportion of patients with ASA physical status III (*p* = 0.010) and coronary artery disease (*p* = 0.009) was significantly higher in the old–old group (Group C). Weight, body mass index, and sex distribution were comparable among the groups. Other comorbidities and baseline vital signs also showed no significant differences across the cohorts. The distribution of surgical procedures differed significantly among the cohorts (*p* = 0.008), with knee/lower-extremity procedures being more frequent in the old–old Group C (85.0%) than in Groups A (45.0%) and B (40.0%). Overall surgical duration was longer in Group C (105 [89–112] min) compared with Group A (78 [71–88] min) and Group B (86 [63–105] min; *p* = 0.034). However, in post hoc procedure-specific subgroup analyses, surgical duration did not differ significantly among age groups for either knee/lower-extremity procedures (*p* = 0.214) or hip arthroplasties (*p* = 0.581). Anesthesia duration (overall *p* = 0.242; knee *p* = 0.326; hip *p* = 0.780) and sedation duration (overall *p* = 0.499; knee *p* = 0.343; hip *p* = 0.396) were also comparable across age cohorts both overall and within procedure subgroups. The median interval from spinal injection to discontinuation of remimazolam was 110 [90–124] min, and that to the end of surgery was 135 [110–148] min, with no differences among groups (*p* = 0.629 and *p* = 0.267, respectively). Surgical procedures, spinal anesthetic doses, achieved sensory blockade levels, adjunctive regional analgesia, together with the distribution of sedation failures, are summarized in Supplementary Table S2.

### 3.2. Maintenance ED_50_ and Exploratory ED_80_, ED_85_, and ED_90_ of Remimazolam

**Figure 2:**
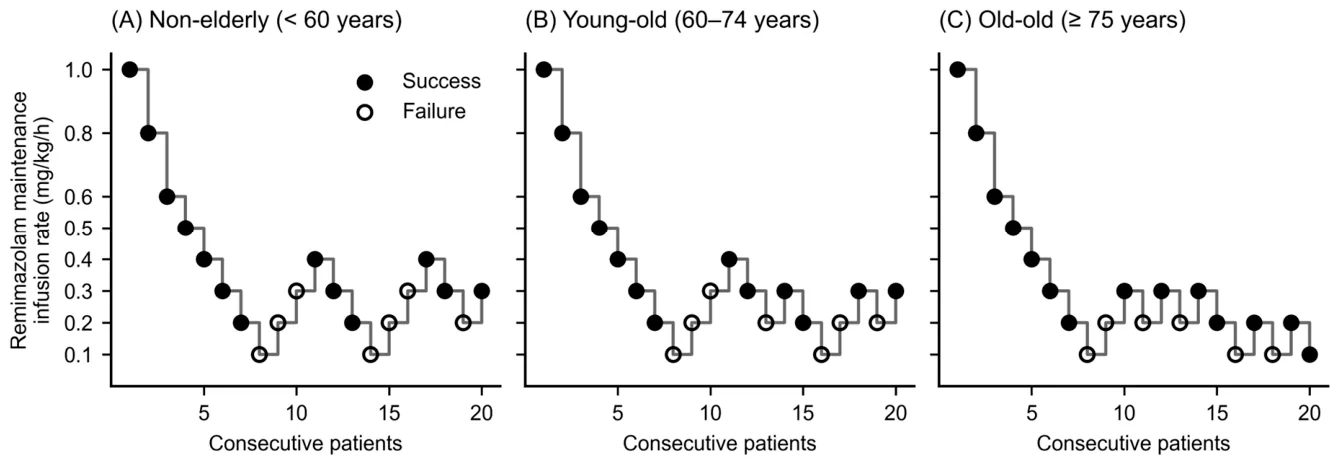
Sequential up-and-down allocation of remimazolam maintenance infusion rates. The sequential trajectories of remimazolam maintenance infusion rates (mg/kg/h) are shown chronologically for 20 consecutive patients in (**A**) Group A (non-elderly, <60 years), (**B**) Group B (young–old, 60–74 years), and (**C**) Group C (old–old, ≥75 years). Solid circles represent successful sedation maintenance (defined as maintaining an mOAA/S score of ≤3 without requiring incremental dose titration throughout the surgery). Open circles represent failure of sedation maintenance (defined as an mOAA/S score > 3 requiring incremental dose titration).

The sequential up-and-down allocation sequences for each group are shown in Figure 2. Successful sedation maintenance was achieved in 13 of 20 patients (65.0%) in Group A, 13 of 20 (65.0%) in Group B, and 14 of 20 (70.0%) in Group C. At the completion of enrollment, the numbers of independent failure-to-success crossover pairs were 3, 4, and 5 in Groups A, B, and C, respectively.

All 20 sedation failures (Group A, *n* = 7; Group B, *n* = 7; Group C, *n* = 6) met the prespecified failure criterion of inadequate sedation (mOAA/S score 4 or 5) during the maintenance phase; the maximum mOAA/S score was 5 in 17 patients and 4 in 3 patients. Failure occurred at a median of 28.5 [15.0–40.8] min after the start of remimazolam administration (Group A: 25.0 [15.5–40.0] min; Group B: 18.0 [15.5–38.5] min; Group C: 38.5 [18.5–42.0] min; *p* = 0.993). In all failures, adequate sedation (mOAA/S ≤ 3) was restored by upward titration of the maintenance infusion rate, with a median cumulative increase of 0.5 [0.5–0.6] mg/kg/h (Group A: 0.6 [0.5–0.8]; Group B: 0.5 [0.5–0.6]; Group C: 0.5 [0.4–0.5] mg/kg/h; *p* = 0.292). Sedation failures occurred in 14 of 26 patients undergoing hip arthroplasty and in 6 of 34 patients undergoing knee or other lower-extremity procedures (Supplementary Table S2).

**Figure 3:**
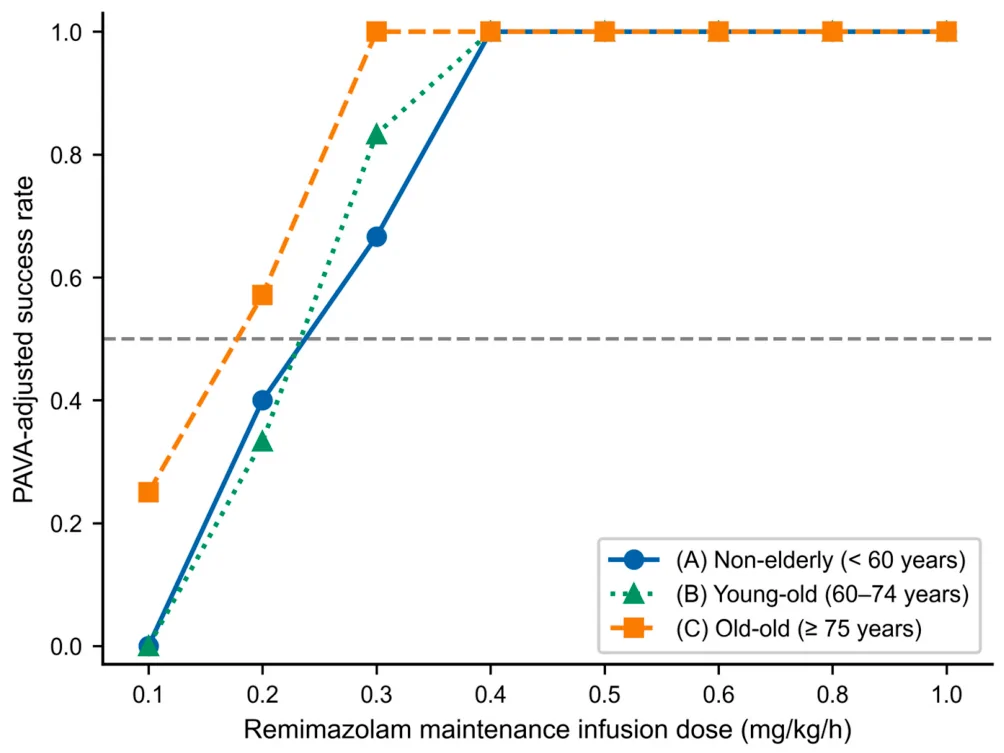
Pooled-adjacent-violators algorithm (PAVA)-adjusted success rate of remimazolam maintenance infusion. The PAVA-adjusted probability of successful sedation maintenance (*y*-axis) plotted against the remimazolam maintenance infusion dose (*x*-axis) for Group A (non-elderly, <60 years; blue solid line with circles), Group B (young–old, 60–74 years; green dotted line with triangles), and Group C (old–old, ≥75 years; orange dashed line with squares). The horizontal dashed line represents the 50% response probability (probability of 0.5) used to determine the median effective dose (ED_50_) for each cohort.

The estimated continuous remimazolam maintenance ED_50_ determined by primary CIR was 0.178 mg/kg/h (90% CI, 0.092–0.245) in the old–old group (Group C, ≥75 years), 0.233 mg/kg/h (90% CI, 0.170–0.301) in the young–old group (Group B, 60–74 years), and 0.238 mg/kg/h (90% CI, 0.152–0.336) in the non-elderly group (Group A, <60 years) (Table 2, Figure 3). The 90% confidence intervals for these estimates overlapped among all three age groups, and no statistically significant difference was observed. Sensitivity analyses using two-parameter log-logistic regression models yielded consistent ED_50_ estimates of 0.159 mg/kg/h, 0.226 mg/kg/h, and 0.233 mg/kg/h for Groups C, B, and A, respectively (Figure S1).

The ED_50_ values overlapped at their 90% CIs, and no significant differences were observed among the groups. CI, confidence interval; ED_50_, median effective dose; *n* (S/F), number of patients (successful/failed sedation).

High-percentile effective doses (ED_80_, ED_85_, and ED_90_) estimated via CIR are presented in Table S1. The estimated ED_90_ was 0.370 (90% CI, 0.347–0.930), 0.340 (90% CI, 0.322–1.230), and 0.277 (90% CI, 0.261–0.561) mg/kg/h in Groups A, B, and C, respectively (Table S1). The 90% confidence intervals for high-percentile estimates overlapped extensively across all three age groups. These intervals were markedly asymmetric, with wide upper limits (exceeding the highest tested dose in Group B), reflecting the sparse observations above the ED_50_ in median-targeted up-and-down designs.

### 3.3. Hemodynamic, Respiratory and Intraoperative Clinical Events

**Figure 4:**
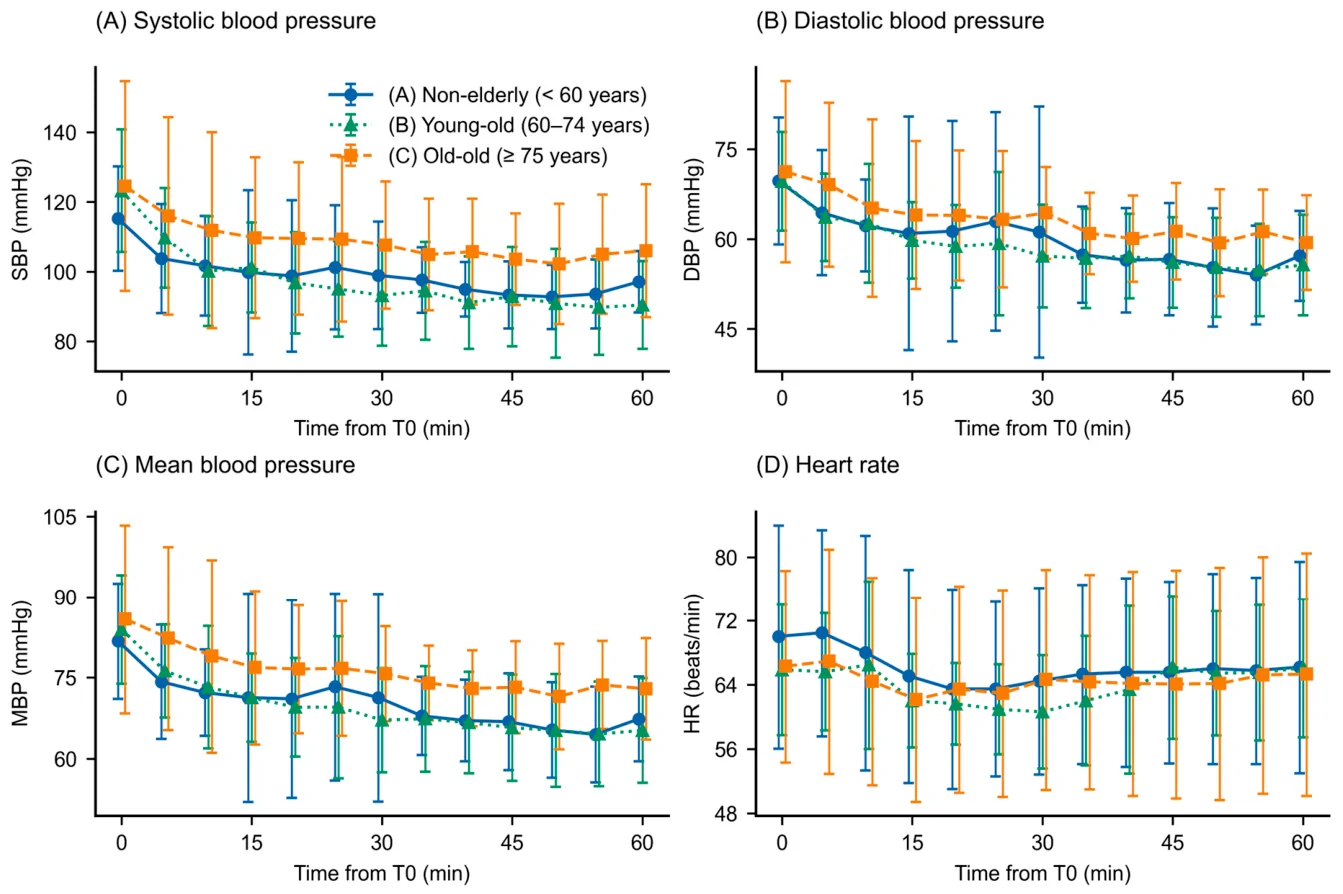
Time courses of perioperative hemodynamic parameters. Changes in (**A**) systolic blood pressure (SBP), (**B**) diastolic blood pressure (DBP), (**C**) mean blood pressure (MBP), and (**D**) heart rate (HR) are plotted over time across the three age groups (Group A: blue solid line with circles; Group B: green dotted line with triangles; Group C: orange dashed line with squares). T0 represents the baseline value immediately before starting the remimazolam loading infusion. Values are presented as mean ± standard deviation (SD) for all patients (N = 60). No clinically significant hemodynamic differences were observed among the three groups.

Perioperative changes in blood pressure and heart rate are shown in Figure 4, and intraoperative hemodynamic and respiratory events evaluated across the full cohorts are summarized in Table 3. Hypotension requiring vasopressor support occurred in 15 of 60 patients (25.0% overall; Group A: 4/20 [20.0%], Group B: 7/20 [35.0%], Group C: 4/20 [20.0%]; *p* = 0.602). All 15 patients received ephedrine, and 5 of them (8.3%) additionally received phenylephrine. No patient required atropine for bradycardia. Respiratory depression requiring airway management occurred in 14 of 60 patients (23.3% overall; Group A: 2/20 [10.0%], Group B: 6/20 [30.0%], Group C: 6/20 [30.0%]; *p* = 0.256). Compared with Group A, the risk differences for hypotension were 15.0% (95% CI, −12.4% to 39.8%) in Group B and 0.0% (95% CI, −24.7% to 24.7%) in Group C, and those for respiratory depression were 20.0% (95% CI, −5.4% to 43.1%) in both Groups B and C. Risk differences with 95% CIs for all intraoperative events are provided in Table S3; all 95% CIs included zero. Airway interventions included head tilt or chin lift in 4 patients (6.7%) and airway insertion in 10 patients (16.7%). SpO_2_ ≤ 95% was observed in 14 patients (23.3% overall; Group A: 2/20 [10.0%], Group B: 5/20 [25.0%], Group C: 7/20 [35.0%]; *p* = 0.207), but no patient experienced severe hypoxemia (SpO_2_ < 90%) or required pharmacological reversal with flumazenil.

### 3.4. Onset, Emergence, and Pharmacodynamic Indicators (PSI and C_e_)

Characteristics of onset, emergence, PSI values, and simulated C_e_ across key landmarks are presented in Table 4. Time to loss of responsiveness (LOR) (Group A: 262 [218–283] s, Group B: 238 [210–252] s, Group C: 210 [187–254] s; *p* = 0.201) and total remimazolam dose required until LOR (Group A: 5.03 [3.92–5.85] mg, Group B: 4.44 [3.79–5.11] mg, Group C: 3.63 [3.18–4.97] mg; *p* = 0.136) showed no significant differences among groups. Time to recovery from drug cessation was also comparable (Group A: 776 ± 414 s, Group B: 950 ± 489 s, Group C: 902 ± 326 s; *p* = 0.564).

PSI values showed no significant differences among age groups across any landmark timepoints (baseline, LOR, drug cessation, or ROR; all *p* > 0.10). In exploratory analyses, the modeled C_e_ exhibited a numerical downward trend with advancing age at LOR (*p* = 0.056), whereas C_e_ at ROR was lower in Group C (0.10 [0.07–0.15] µg/mL) compared with Group B (0.17 [0.10–0.18] µg/mL) and Group A (0.17 [0.11–0.24] µg/mL; *p* = 0.007). Additionally, C_e_ at drug cessation was analyzed exclusively in patients with successful sedation, because the maintenance rate had been titrated in sedation failures and drug cessation under the assigned rate was therefore not applicable. In some patients, PSI data were unavailable or unreliable because of occasional technical malfunction of the monitor.

## 4. Discussion

To our knowledge, this is the first prospective study to estimate age-stratified continuous maintenance ED_50_ values of remimazolam for moderate sedation during spinal anesthesia; age-related differences in remimazolam requirements have previously been reported mainly for induction or loss of consciousness during general anesthesia [9,10,11], and the randomized sedation trials were conducted in endoscopic and bronchoscopic procedures [17,18,19]. The maintenance ED_50_ was numerically lower in the old–old cohort (0.178 mg/kg/h) than in the non-elderly (0.238 mg/kg/h) and young–old (0.233 mg/kg/h) cohorts. However, the 90% confidence intervals overlapped substantially, and our primary analysis did not demonstrate a statistically significant age-dependent difference. With 20 patients per group, the study was not powered to detect a difference of this magnitude. These estimates should therefore be regarded as hypothesis-generating reference values for initial maintenance titration rather than as definitive dosing rules, and the lower point estimate in the oldest cohort provides, at most, a safety-motivated rationale for cautious individualized titration in this population.

Previous dose-finding studies of remimazolam under regional anesthesia have mainly evaluated single-bolus doses or loading infusions [20,21]. For maintenance, Qiao et al. used an average infusion rate of 0.223 mg/kg/h to maintain deeper sedation (mOAA/S ≤ 2) in elderly patients under neuraxial anesthesia [22]. Our maintenance ED_50_ estimates are of a similar magnitude, although such cross-study comparisons involve different endpoints and populations and should be interpreted with caution. Exploratory higher-percentile estimates (ED_80_–ED_90_) also showed a numerical decline with age, but their wide and overlapping confidence intervals reflect the limited information that median-targeted up-and-down designs provide at the extremes of the dose–response curve; they are therefore not decision-grade targets [12,16]. Group C included more knee procedures and had a longer overall surgical duration. Although surgical and sedation durations did not differ among age groups within procedure categories, these subgroup comparisons were small, and residual confounding by procedure type and comorbidity cannot be excluded.

Intraoperative events were evaluated in the full cohort (N = 60). Hypotension requiring vasopressor support occurred in 25.0% of patients and respiratory depression requiring airway management in 23.3%, without statistically significant differences among age groups (*p* = 0.602 and *p* = 0.256, respectively), and no patient required atropine for bradycardia (Table 3). Respiratory events were managed with head tilt or chin lift (*n* = 4) or airway insertion (*n* = 10); no patient had SpO_2_ below 90% or required flumazenil. Nevertheless, respiratory depression occurred in 30.0% of patients in each of the two older groups. Under regional anesthesia, a retrospective study of orthopedic surgery reported capnography-defined apnea (expired CO_2_ < 1 mmHg for ≥10 s) in 63.9% of patients receiving continuous remimazolam [23]; although this definition differs from ours, these data indicate that respiratory events are common during continuous remimazolam sedation. These findings indicate that the adverse events observed in our study were manageable but not infrequent, and that careful hemodynamic and airway monitoring remains necessary, particularly in older patients.

Time to LOR, the loading dose required to reach LOR, and the time from drug cessation to ROR were comparable across age groups (Table 4), consistent with the short context-sensitive half-life and esterase-based metabolism of remimazolam [5,6]. PSI values did not differ significantly among groups at any landmark, although PSI data were incomplete at some landmarks. In exploratory analyses, simulated C_e_ showed a numerical downward trend with advancing age at LOR (0.17 µg/mL in Group C vs. 0.21–0.23 µg/mL in Groups A and B; *p* = 0.056) and was lower in Group C at ROR (0.10 vs. 0.17 µg/mL; *p* = 0.007). Previous studies using target-controlled infusion have also reported lower remimazolam C_e_ with advancing age [24,25]. However, our C_e_ values were derived post hoc from the Schüttler model developed in healthy male volunteers [14,15] rather than measured, and these exploratory comparisons were not adjusted for multiplicity. They should therefore be interpreted as compatible with, but not demonstrating, increased pharmacodynamic sensitivity in the oldest patients.

Several limitations of this study warrant consideration. First, because the initial sequence in each cohort underwent a consecutive descent before reaching the target oscillation zone, fewer patients were allocated directly around the ED_50_, contributing to higher overall success proportions and wider confidence intervals for tail percentiles; thus, higher-percentile estimates should be regarded as exploratory, and no pre-study simulation of the step sizes and stopping rule was performed. In addition, the number of failure-to-success crossover pairs (3–5 per cohort) was lower than the planned six, which is reflected in the width of the confidence intervals. Second, sedation success was evaluated through surgical completion rather than over a fixed-time observation window. Surgical procedures follow standardized techniques in our hospital with predictable durations (typically within 2 h), and intrathecal bupivacaine with fentanyl was expected to provide adequate anesthesia throughout surgery [1,26]; however, continuous intraoperative or postoperative reassessment of sensory block levels was not conducted, which could have provided tighter verification of block depth versus late sedation requirements. In addition, residual drug from the standardized loading infusion may have contributed to early maintenance sedation. Third, baseline differences in surgical procedure types, adjunctive regional analgesia, ASA physical status, and comorbidities existed among cohorts; age and ASA physical status have both been identified as covariates of remimazolam pharmacokinetics [27]. These unadjusted baseline clinical, surgical and anesthetic imbalances may still have influenced the observed numerical age trend, limiting the ability to attribute dosing differences solely to age. Additionally, this was a single-center, open-label trial without an active comparator group; the attending anesthesiologists were aware of the assigned infusion rate, and our findings in patients with ASA physical status I–III undergoing lower-extremity surgery may not directly apply to higher-risk populations or non-orthopedic procedures.

## 5. Conclusions

In conclusion, the continuous maintenance ED_50_ of remimazolam for moderate sedation during spinal anesthesia was numerically lower in old–old patients (aged ≥75 years), without a statistically significant difference among age groups. Hypotension and respiratory events requiring intervention were not infrequent but were manageable with vasopressors and simple airway interventions. Based on safety considerations, a conservative initial maintenance rate with individualized titration may be considered in patients aged ≥75 years; this hypothesis-generating suggestion requires confirmation in adequately powered, comparative studies.

## Figures and Tables

**Table 1:** Demographic and baseline characteristics.

Characteristic	A. Non-Elderly (<60) (*n* = 20)	B. Young–Old (60–74) (*n* = 20)	C. Old–Old (≥75) (*n* = 20)	*p*
Age (years)	51.5 [45.0–54.2]	66.0 [63.8–69.5]	79.0 [76.0–81.0]	<0.001
Sex (male/female)	8/12	9/11	5/15	0.393
Height (m)	1.62 [1.57–1.72]	1.59 [1.56–1.63]	1.53 [1.49–1.56]	0.001
Weight (kg)	69.7 ± 15.0	65.7 ± 9.4	63.4 ± 8.4	0.216
BMI (kg/m^2^)	25.9 ± 4.5	25.6 ± 2.8	26.9 ± 3.1	0.497
ASA physical status, *n* (%)				0.010
I	5 (25.0)	2 (10.0)	0 (0.0)	
II	15 (75.0)	17 (85.0)	15 (75.0)	
III	0 (0.0)	1 (5.0)	5 (25.0)	
Comorbidities, *n* (%)				
Diabetes mellitus	2 (10.0)	6 (30.0)	8 (40.0)	0.092
Hypertension	6 (30.0)	9 (45.0)	13 (65.0)	0.084
CAD	0 (0.0)	0 (0.0)	5 (25.0)	0.009
CVA	0 (0.0)	0 (0.0)	1 (5.0)	1.000
Surgery type, *n* (%)				0.008
Knee/LE procedures	9 (45.0)	8 (40.0)	17 (85.0)	
Hip	11 (55.0)	12 (60.0)	3 (15.0)	
Anesthesia duration (min)				
Overall	136 [119–148]	150 [118–156]	148 [138–164]	0.242
Knee/LE procedures	119 [103–160]	164 [148–178]	152 [138–165]	0.326
Hip arthroplasty	138 [130–146]	127 [116–153]	140 [134–140]	0.780
Surgical duration (min)				
Overall	78 [71–88]	86 [63–105]	105 [89–112]	0.034
Knee/LE procedures	77 [54–101]	114 [89–124]	108 [97–112]	0.214
Hip arthroplasty	78 [75–82]	72 [60–87]	77 [76–78]	0.581
Sedation duration (min)				
Overall	85.0 [75.5–103.5]	82.0 [74.8–106.2]	98.0 [89.0–102.2]	0.499
Knee/LE procedures	79.0 [60.0–85.0]	94.5 [74.5–110.5]	100.0 [86.0–103.0]	0.343
Hip arthroplasty	97.5 [84.0–104.3]	79.5 [74.7–99.0]	97.0 [94.5–98.0]	0.396
Baseline vital signs				
SBP (mmHg)	112.0 [107.0–122.8]	124.0 [111.5–132.5]	120.5 [104.5–137.8]	0.338
DBP (mmHg)	71.5 [65.8–74.2]	71.0 [64.5–73.0]	72.0 [62.8–79.5]	0.712
MBP (mmHg)	81.5 [78.5–85.0]	84.5 [77.2–89.2]	85.0 [78.2–90.2]	0.503
HR (beats/min)	69.0 [62.5–83.0]	65.0 [60.8–72.8]	64.5 [58.0–69.5]	0.406
RR (breaths/min)	13.8 ± 3.7	14.6 ± 3.1	15.0 ± 3.2	0.558
SpO_2_ (%)	100.0 [99.0–100.0]	99.0 [98.0–100.0]	99.5 [99.0–100.0]	0.697

Values are median [IQR], mean ± SD, or *n* (%). *p* values for normally distributed continuous variables were calculated using one-way analysis of variance and those for non-normally distributed continuous variables using the Kruskal–Wallis test; *p* values for categorical variables were calculated using the chi-square test or Fisher’s exact test. ASA, American Society of Anesthesiologists; BMI, body mass index; CAD, coronary artery disease; CVA, cerebrovascular accident; DBP, diastolic blood pressure; HR, heart rate; IQR, interquartile range; LE, lower extremity; MBP, mean blood pressure; RR, respiratory rate; SBP, systolic blood pressure; SD, standard deviation; SpO_2_, peripheral oxygen saturation.

**Table 2:** The ED_50_ with 90% CI of remimazolam maintenance infusion for successful sedation using centered isotonic regression, by age group.

Age Group	*n* (S/F)	ED_50_ (mg/kg/h)	90% CI
A. Non-elderly (<60)	20 (13/7)	0.238	(0.152–0.336)
B. Young–old (60–74)	20 (13/7)	0.233	(0.170–0.301)
C. Old–old (≥75)	20 (14/6)	0.178	(0.092–0.245)

**Table 3:** Intraoperative hemodynamic and respiratory data.

Event, *n* (%)	Overall (*n* = 60)	A (<60) (*n* = 20)	B (60–74) (*n* = 20)	C (≥75) (*n* = 20)	*p*
Hypotension requiring vasopressor	15 (25.0)	4 (20.0)	7 (35.0)	4 (20.0)	0.602
Ephedrine	15 (25.0)	4 (20.0)	7 (35.0)	4 (20.0)	
Phenylephrine	5 (8.3)	2 (10.0)	0 (0.0)	3 (15.0)	
Bradycardia requiring atropine	0 (0.0)	0 (0.0)	0 (0.0)	0 (0.0)	–
Respiratory depression requiring airway management	14 (23.3)	2 (10.0)	6 (30.0)	6 (30.0)	0.256
Head tilt or chin lift	4 (6.7)	1 (5.0)	1 (5.0)	2 (10.0)	
Airway insertion	10 (16.7)	1 (5.0)	5 (25.0)	4 (20.0)	
Flumazenil administration	0 (0.0)	0 (0.0)	0 (0.0)	0 (0.0)	
SpO_2_ < 90%	0 (0.0)	0 (0.0)	0 (0.0)	0 (0.0)	–
SpO_2_ ≤ 95%	14 (23.3)	2 (10.0)	5 (25.0)	7 (35.0)	0.207

Values are *n* (%). *p* values were calculated using Fisher’s exact test; a dash (–) indicates no events. All patients with hypotension received ephedrine; 5 patients (Group A, *n* = 2; Group C, *n* = 3) additionally received phenylephrine. SpO_2_, peripheral oxygen saturation.

**Table 4:** PSI and C_e_ of remimazolam, by age group.

Variable	A (<60) (*n* = 20, S/F 13/7)	B (60–74) (*n* = 20, S/F 13/7)	C (≥75) (*n* = 20, S/F 14/6)	*p*
Onset				
Time to LOR, s	262 [218–283]	238 [210–252]	210 [187–254]	0.201
Remimazolam dose until LOR, mg	5.03 [3.92–5.85]	4.44 [3.79–5.11]	3.63 [3.18–4.97]	0.136
Recovery				
Time from drug cessation to ROR, s	776 ± 414 (*n* = 13)	950 ± 489 (*n* = 12)	902 ± 326 (*n* = 11)	0.564
PSI				
Baseline	90 [89–92]	94 [89–94] (*n* = 16)	89 [88–93] (*n* = 15)	0.306
LOR	78 [64–84] (*n* = 19)	72 [64–80] (*n* = 16)	79 [74–85] (*n* = 15)	0.267
Drug cessation	32 [26–40] (*n* = 13)	26 [21–33] (*n* = 8)	46 [34–54] (*n* = 8)	0.123
ROR	74 [68–78]	76 [62–83] (*n* = 14)	79 [67–84] (*n* = 13)	0.622
C_e_ (µg/mL)				
LOR	0.23 [0.19–0.26]	0.21 [0.18–0.24]	0.17 [0.16–0.19]	0.056
Drug cessation	0.35 [0.28–0.42] (*n* = 13)	0.30 [0.23–0.47] (*n* = 13)	0.24 [0.20–0.40] (*n* = 14)	0.419
ROR	0.17 [0.11–0.24]	0.17 [0.10–0.18] (*n* = 19)	0.10 [0.07–0.15] (*n* = 17)	0.007

Data are median [IQR] or mean ± SD (*n*). *p* values were calculated using the Kruskal–Wallis test or one-way analysis of variance. Numbers of measurements differ across landmarks and groups. PSI values were unavailable in some patients because of occasional technical malfunction of the PSI monitor. C_e_ at ROR could not be simulated in 4 patients (Group B, *n* = 1; Group C, *n* = 3) because the ROR timepoint required for the simulation was not accurately recorded. Separately, the time from drug cessation to ROR was not available in 4 patients with successful sedation (Group B, *n* = 1; Group C, *n* = 3). C_e_ at drug cessation and time from drug cessation to ROR are shown for patients with successful sedation only. C_e_, effect-site concentration; F, failure; IQR, interquartile range; LOR, loss of responsiveness; PSI, Patient State Index; ROR, recovery of responsiveness; SD, standard deviation; S, success.

## Data Availability

The de-identified data and the R analysis code supporting the findings of this study are available from the corresponding author upon reasonable request. The data are not publicly available because public release of patient-level clinical data is restricted by institutional privacy regulations.

## Supplementary Materials

The following supporting information can be downloaded at: https://www.mdpi.com/article/10.3390/medicina62101937/s1, Figure S1: Comparison of centered isotonic regression and log-logistic regression models; Table S1: Exploratory ED_80_, ED_85_, and ED_90_ of remimazolam maintenance infusion with 90% confidence intervals, by age group; Table S2: Surgical procedures and anesthetic approach by age group; Table S3: Risk differences with 95% confidence intervals for intraoperative hemodynamic and respiratory events relative to Group A.

## Abbreviations

The following abbreviations are used in this manuscript:

ASA	American Society of Anesthesiologists
C_e_	Effect-site concentration
CI	Confidence interval
CIR	Centered isotonic regression
ED_50_	Median effective dose
ED_80_, ED_85_, ED_90_	80%, 85%, and 90% effective doses
HR	Heart rate
IQR	Interquartile range
LOR	Loss of responsiveness
MBP	Mean blood pressure
mOAA/S	Modified Observer’s Assessment of Alertness/Sedation
PAVA	Pooled-adjacent-violators algorithm
PSI	Patient State Index
ROR	Recovery of responsiveness
RR	Respiratory rate
SD	Standard deviation
SpO_2_	Peripheral oxygen saturation
