# Supplementary material for: Age-Stratified Median Effective Dose of a Continuous Remimazolam Maintenance Infusion for Moderate Sedation During Spinal Anesthesia: A Prospective Up-and-Down Dose-Finding Study
Source: Medicina (Kaunas). 2026 Oct 8;62(10):1937. doi: 10.3390/medicina62101937 (PMC13707296; doi:10.3390/medicina62101937)
Supplement: Supplementary file 1 [file medicina-62-01937-s001.zip › medicina-4563924-supplementary.pdf]

Supplementary Materials

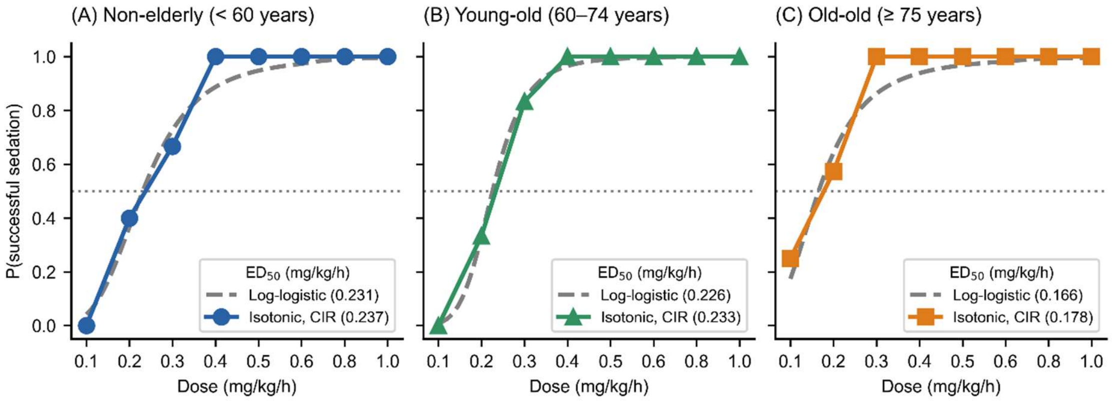

Figure S1. Comparison of centered isotonic regression and log-logistic regression models. Fitted dose-response curves predicting the probability of successful sedation maintenance across the three cohorts: (A) Group A (non-elderly, < 60 years), (B) Group B (young-old, 60–74 years), and (C) Group C (old-old, ≥ 75 years). The solid lines with marker symbols represent the primary centered isotonic regression (CIR) curves, and the grey dashed lines represent the fitted two-parameter log-logistic regression curves used as sensitivity analyses. The estimated ED_{50} value (mg/kg/h) for each model is shown in parentheses in the legends. The horizontal dotted line represents the 50% response probability

Table S1. Exploratory ED_{80}, ED_{85}, and ED_{90} of remimazolam maintenance infusion with 90% confidence intervals, by age group.

Age group	*n* (S/F)	ED_{80} (90% CI)	ED_{85} (90% CI)	ED_{90} (90% CI)
A. Non-elderly (< 60)	20 (13/7)	0.340 (0.293–0.687)	0.355 (0.320–0.809)	0.370 (0.347–0.930)
B. Young-old (60–74)	20 (13/7)	0.293 (0.265–0.410)	0.310 (0.285–0.646)	0.340 (0.322–1.230)
C. Old-old (≥ 75)	20 (14/6)	0.253 (0.222–0.467)	0.265 (0.242–0.514)	0.277 (0.261–0.561)

All doses are expressed in mg/kg/h. Estimates were obtained by centered isotonic regression with analytic 90% CIs and are exploratory; the intervals are asymmetric because observations above the ED50 are sparse in median-targeted up-and-down designs, and the upper limit in Group B exceeds the highest tested dose (1.0 mg/kg/h). CI, confidence interval; ED, effective dose; *n* (S/F), number of patients (successful/failed sedation).

Table S2. Surgical procedures and anesthetic approach by age group.

Characteristic	A (< 60) (*n* = 20)	B (60–74) (*n* = 20)	C (≥ 75) (*n* = 20)
**Spinal anesthesia**			
0.5% hyperbaric bupivacaine (mg), median [IQR]	13.0 [13.0–14.0]	13.0 [13.0–14.0]	13.0 [12.4–13.0]
Intrathecal fentanyl (μg)	15	15	15
Achieved sensory block level †, *n* (failures)			
T4	1 (0)	0	0
T8	0	0	2 (0)
T10	18 (6)	17 (7)	14 (5)
Not recorded	1 (1)	3 (0)	4 (1)

Characteristic	A (< 60) (*n* = 20)	B (60–74) (*n* = 20)	C (≥ 75) (*n* = 20)
**Surgical procedure and adjunctive regional analgesia, *n* (failures)**			
**Hip arthroplasty (THRA)**	11 (6)	12 (7)	3 (1)
FICB	10 (6)	12 (7)	3 (1)
QL block	1 (0)	0	0
**Knee or other lower-extremity procedure**	9 (1)	8 (0)	17 (5)
TKRA, spinal anesthesia alone	0	7 (0)	11 (4)
TKRA + femoral nerve block	0	0	1 (0)
Revision TKRA + epidural supplementation	0	0	1 (0)
Unicompartmental knee arthroplasty	1 (0)	0	2 (0)
High tibial osteotomy	3 (1)	0	0
Arthroscopic or cartilage-restoration knee procedure	5 (0)	0	0
Open implant removal, knee	0	1 (0)	0
ORIF of patella fracture + femoral nerve block	0	0	1 (1)
Implanted device removal, tibia	0	0	1 (0)

Values are number of patients or median [IQR]. Numbers in parentheses indicate patients with sedation failure (mOAA/S score > 3 requiring dose titration) within each category. † Sensory block levels were documented in 52 patients. FICB, fascia iliaca compartment block; IQR, interquartile range; mOAA/S, Modified Observer's Assessment of Alertness/Sedation; ORIF, open reduction and internal fixation; QL, quadratus lumborum; THRA, total hip replacement arthroplasty; TKRA, total knee replacement arthroplasty.

Table S3. Risk differences with 95% confidence intervals for intraoperative hemodynamic and respiratory events relative to Group A.

Event	A (< 60) (*n* = 20)	B (60–74) (*n* = 20)	C (≥ 75) (*n* = 20)	RD, B vs. A (95% CI)	RD, C vs. A (95% CI)
Hypotension requiring vasopressor	4 (20.0)	7 (35.0)	4 (20.0)	15.0 (−12.4 to 39.8)	0.0 (−24.7 to 24.7)
Ephedrine	4 (20.0)	7 (35.0)	4 (20.0)	15.0 (−12.4 to 39.8)	0.0 (−24.7 to 24.7)
Phenylephrine	2 (10.0)	0 (0.0)	3 (15.0)	−10.0 (−30.1 to 7.7)	5.0 (−17.3 to 27.2)
Bradycardia requiring atropine	0 (0.0)	0 (0.0)	0 (0.0)	0.0 (−16.1 to 16.1)	0.0 (−16.1 to 16.1)
Respiratory depression requiring airway management	2 (10.0)	6 (30.0)	6 (30.0)	20.0 (−5.4 to 43.1)	20.0 (−5.4 to 43.1)
Head tilt or chin lift	1 (5.0)	1 (5.0)	2 (10.0)	0.0 (−19.1 to 19.1)	5.0 (−15.0 to 25.5)
Airway insertion	1 (5.0)	5 (25.0)	4 (20.0)	20.0 (−3.2 to 42.3)	15.0 (−7.1 to 37.0)
Flumazenil administration	0 (0.0)	0 (0.0)	0 (0.0)	0.0 (−16.1 to 16.1)	0.0 (−16.1 to 16.1)
SpO_2 < 90%	0 (0.0)	0 (0.0)	0 (0.0)	0.0 (−16.1 to 16.1)	0.0 (−16.1 to 16.1)
SpO_2 ≤ 95%	2 (10.0)	5 (25.0)	7 (35.0)	15.0 (−9.4 to 38.0)	25.0 (−1.3 to 47.9)

Values are *n* (%) or risk difference in percentage points (95% CI). Risk differences relative to Group A were calculated using the Newcombe hybrid score method. All patients with hypotension received ephedrine; 5 patients (Group A, *n* = 2; Group C, *n* = 3) additionally received phenylephrine. CI, confidence interval; RD, risk difference; SpO_2, peripheral oxygen saturation.
